# Machine learning in biosignals processing for mental health: A narrative review

**DOI:** 10.3389/fpsyg.2022.1066317

**Published:** 2023-01-13

**Authors:** Elena Sajno, Sabrina Bartolotta, Cosimo Tuena, Pietro Cipresso, Elisa Pedroli, Giuseppe Riva

**Affiliations:** ^1^Humane Technology Lab, Università Cattolica del Sacro Cuore, Milan, Italy; ^2^Department of Computer Science, University of Pisa, Pisa, Italy; ^3^ExperienceLab, Università Cattolica del Sacro Cuore, Milan, Italy; ^4^Department of Psychology, Università Cattolica del Sacro Cuore, Milan, Italy; ^5^Applied Technology for Neuro-Psychology Lab, IRCCS Istituto Auxologico Italiano, Milan, Italy; ^6^Department of Psychology, University of Turin, Turin, Italy; ^7^Department of Psychology, eCampus University, Novedrate, Italy

**Keywords:** biosignals, artificial intelligence, machine learning, mental health, neurology, precision medicine, affective computing, brain–computer interfaces

## Abstract

Machine Learning (ML) offers unique and powerful tools for mental health practitioners to improve evidence-based psychological interventions and diagnoses. Indeed, by detecting and analyzing different biosignals, it is possible to differentiate between typical and atypical functioning and to achieve a high level of personalization across all phases of mental health care. This narrative review is aimed at presenting a comprehensive overview of how ML algorithms can be used to infer the psychological states from biosignals. After that, key examples of how they can be used in mental health clinical activity and research are illustrated. A description of the biosignals typically used to infer cognitive and emotional correlates (e.g., EEG and ECG), will be provided, alongside their application in Diagnostic Precision Medicine, Affective Computing, and brain–computer Interfaces. The contents will then focus on challenges and research questions related to ML applied to mental health and biosignals analysis, pointing out the advantages and possible drawbacks connected to the widespread application of AI in the medical/mental health fields. The integration of mental health research and ML data science will facilitate the transition to personalized and effective medicine, and, to do so, it is important that researchers from psychological/ medical disciplines/health care professionals and data scientists all share a common background and vision of the current research.

## 1. Introduction

Conventionally, mental health diagnoses require the health care professional to obtain patient’ information from narrative data (anamnesis and screening tools) and observation. On the one hand, these methodologies have the great advantage of allowing a qualitative assessment of the patients, allowing them to feel at the center of the treatment process and express themself. On the other hand, these methods are highly subjective, and therefore prone to possible incomplete or biased information (Oscar, 2022). Furthermore, self-report tools can take a long time to administer and be time-consuming, leading to inefficiency (Thabtah et al., 2018). These drawbacks can affect the medical journey in all steps, from providing the correct diagnosis to administering effective treatments. To overcome these limitations, there are two possible approaches equally suitable, and which can be implemented complementary: (1) simplify and add information to the self-reported data obtained by the patient; (2) use more objective clinical data, able to inform and monitor all the medical journey of the patient.

For the latter point, Machine Learning (ML) represents an optimal tool to partially overcome the limitations of solely using self-report data, by objectively determining which factors contribute the most to the informativeness of the clinical problem under investigation and integrating new information into the decision-making process. Indeed, ML proved to be effective in several clinical domains. It has been found that ML models enable the selection of those biomarkers that are the most prognostic of antidepressant treatment outcomes, thus overcoming the elusiveness and the complexity of the interrelation between different phenotypes related to depression, which typically do not allow a clinician to predict if a treatment will be effective or not (Athreya et al., 2019). Or, again, ML algorithms allow the interpretation of EEG signals linked to specific brain activity (e.g., the activity of the motor/premotor cortex), to be used as inputs in Brain-Computer Interfaces (BCIs) applications (Birbaumer, 2006) as communicator systems for people with ALS (Graimann et al., 2010).

The ML-based analysis of biosignals would allow a more objective, autonomous, and automatic collection of a patient’s clinical data, providing a detailed picture of a patient’s health status (Oscar, 2022). The vast trade in recent years of ever smaller, wireless, and battery-free biosignal collection tools (Stuart et al., 2021) has resulted in greater advancement of biosensing research applied to the clinical field. Biosignals are physiological measures (e.g., electroencephalography, heart rate) indicative of not only physical states but also mental functions. The clinical applications that ML-based biosignals analysis entails are diverse, such as recognition and classification of abnormal patterns of brain functioning, recognition and classification of various affective and cognitive states, selection of the best treatment strategy for the specific situation, and so on.

Currently, ML models allow the collection and analysis of large amounts of biosignals (i.e., Big Data) that could represent interesting biomarkers of the brain and body functioning (i.e., sympathetic and parasympathetic system activation), and mental processes (Riva et al., 2019). This will enable individual treatment strategies, transition to personalized, effective, and engaging medicine (Riva et al., 2019), and potentially reshape the healthcare system by improving the quality overall of care (Kalantari et al., 2018).

The purpose of this narrative review is to illustrate the ML applications to psychophysiological data analysis, deepening how ML techniques could bring new and interesting knowledge and open up new areas of discussion in the study of human mental health. First, an elucidation of different biosignals typically used to infer psychological correlates is provided, with a description of the relation between physiological data and mental functions (e.g., cognitive, emotional). An explanation of ML models (e.g., supervised, unsupervised), and issues associated with them (e.g., “garbage-in garbage-out”) are presented. Practical applications and experiments are then illustrated, to give the reader a complete overview of the possible clinical implementations of ML-based analysis of biosignals. We provide an in-depth analysis of the three principal areas in which ML analysis of biosignals has been applied to the mental health field: (1) diagnostic precision medicine, (2) Affective Computing and 3) BCIs. For each topic, we selected studies that have had the greatest impact in their field, as well as reviews on promising innovations. Finally, future challenges and research questions related to ML application in mental health areas are deepened.

## 2. Biosignals for mental health

A showcase of the biosignals most commonly used in psychological research and mental health contexts is illustrated in Table 1. They were selected for their informativeness on the person’s physiological activation and allow both to monitor the health status and to infer some psychological correlates. These biosignals are used in a variety of contexts to infer the implicit subject’s responses to external and internal stimuli/changes associated with brain functioning or sympathetic/parasympathetic arousal changes, indicative, among others, of psychological states.

**Table 1:** Description of biosignals more frequently used in mental health with their respective features and psychological correlates.

Biosignals	Features	Correlates
Cardiac signals recorded *via* • Electrocardiogram (ECG) the electrical activity signals of the heart, recorded on the body’s surface • Blood Volume Pulse (BVP) blood volume fluctuations, used analogously to ECG to infer heart activity. HR derived from BVP data has some milliseconds of latency, caused by calculations from the interbeat interval (Peper et al., 2007) and the location in the body’s periphery (e.g., the fingertips) where signals are collected.	Heart Rate (HR) is the measurement of the frequency of the heart activity cycle Zhang and Zhang (2009), counting the number of contractions (beats) the heart makes per minute (Beats per Minute) Peper et al., 2007.	Related to Autonomic Nervous System activity (both parasympathetic and sympathetic) and breathing Kreibig et al. (2007); Pérez et al. (2021). HR rises in response to physical activity, mental activation, and emotions such as suspense and surprise. It is also linked to attentional focus Pérez et al. (2021).
	Heart Rate Variability (HRV) calculates how the time intervals between one heartbeat and another vary, is correlated to variations in neurocardiac functions, and affected by the activity of the autonomic nervous system Shaffer and Ginsberg (2017). HRV features (i.e., frequency) are indicative of an individual’s activation (Malik et al., 1996; Palma and Benarroch (2014): • very low frequency (VLF) - 0 - 0.04 Hz; • low frequency (LF) 0.04–0.15 Hz; • high frequency (HF) 0.15 and 0.4 Hz.	Increases in HRV show activation of the Parasympathetic Nervous System (SNP), while a decrease in HRV is correlated with increased sympathetic activation. Akselrod et al. (1987); Van Ravenswaaij-Arts et al. (1993); Kreibig et al. (2007); Peper et al. (2007) Findings link the HF components to SNP activity and the LF components to both the Sympathetic Nervous System (SNS) activity and to vagal’s nerve influences Akselrod et al. (1987); Malik et al. (1996); Kreibig et al. (2007); Palma and Benarroch (2014).
Respiratory Signals (RSPS) are measured through rhythm and depth of breath	RSPS contain four-phase cycles Boiten et al. (1994): • inspiratory flow; • inspiratory pause; • expiratory flow; • expiratory pause.	Stress can be detected by longer expiration times and shorter pause times. Meanwhile, other breathing parameters, such as frequency, amplitude, regularity, sighs, tremors, and thoracic tension, have been found to be emotionally distinct Boiten et al. (1994); Malik et al. (1996); Kreibig et al. (2007); Shan et al. (2020). Decreasing breathing frequency decreases HR (Cooke et al., 1998)
	Respiratory Sinus Arrhythmia (RSA) links respiration patterns to heart activity. RSA is characterized by an increase in HR during inspiration and a decrease during expiration. It shows influences on HRV (Giggins et al., 2013; Palma and Benarroch, 2014), and reaches its maximum level with 6 complete breaths per minute, where resonance between respiration and HR oscillations is reached (called Cardiac Coherence; van Ravenswaaij-Arts et al., 1993; Blum et al., 2019)	Slow-paced respiration boosts HRV and could be used for increasing relaxation Blum et al. (2019). Distance between beats (R–R interval) and arterial pressure spectral power increase as breathing frequency decreases Cooke et al. (1998)
Temperature is measured with a “thermistor,” a thermal sensor.	The thermal sensor with a temperature range of about 24–35° C is typically placed on the skin of the palmar surface of one finger, which temperature ranges from about 24 to 35° C (Yucha and Montgomery (2008). BVP can also be used to infer changes in peripheral temperature (Peper et al. (2007).	A rising in peripheral temperature, if not influenced by the environment temperature, correlates with general relaxation, while a lowering is linked to activation (Yucha and Montgomery, 2008 Stress correlates with temperature differently depending on the position on the body (e.g., temperature decreases on the fingertips) (Vinkers et al. (2013) Slower and diaphragmatic breathing helps increase peripheral temperature (Peper et al., 2007.
Electromyography (EMG) allows for the recording of electrical signals in muscles, which is proportional to the degree of contraction of the muscle itself.	The information about muscle contractions is primarily used by physical therapists for muscular and neurological rehabilitation, as well as to retrieve precise data on muscular activation (Yucha and Montgomery (2008). Useful inferences for Mental Health can be deducted by facial muscular activation (i.e., facial expressions), which is strongly related to emotional state (Ekman and Rosenberg (2005)	Activity of corrugator supercilii and zygomatic muscles are considered a reliable indicator of emotional states (Ekman and Rosenberg, 2005; Kreibig et al., 2007; Gromala et al., 2015). Specifically, zygomaticus major and orbicularis oculi relate to positive emotions, whereas corrugator supercilii with negative ones (Cacioppo et al. (2000).
Electrodermal Activity (EDA) or Skin Conductance (SC) also known as “skin conductance activity,” or “galvanic skin response,” is calculated by detecting the electrical conductance of the skin between two sensors, placed on the palmar surface of two fingers, or in two places on the palm Yucha and Montgomery (2008).	EDA/SC components: - Background activity: Tonic components/SC Level; - Sympathetic neuronal activation: Rapid Phasic components/SC Responses. Braithwaite et al. (2013)	Sweat gland activity causes changes in conductivity, which correlates with SNS activity: SC rises with negative emotions such as fear, worry, sadness, or anger, and falls as the subject relaxes Yucha and Montgomery (2008); Pérez et al. (2021).
Electroencephalogram (EEG) detects electrical activity (expressed in microvolts) of the brain’s cortical neurons *via* sensors applied to the scalp with conductive paste.	EEG is analyzed in terms of amplitude and synchronicity in both the time and frequency domains. EEG raw signal is usually subdivided by frequency (through methods like Fast Fourier transformation), facilitating the interpretation of brain activity and its psychological correlates (Chapin and Russell-Chapin, 2013; Al-Fahoum and Al-Fraihat, 2014).	EEG is frequently used to measure Event-Related Potentials (ERPs), defined as stereotyped electrophysiological responses strictly time-locked to a stimulus (Sur and Sinha, 2009). ML algorithms have been successfully used to analyze acoustic ERPs for the early detection of schizophrenia disorder (Frick et al., 2021). ML has been also optimally used to analyze ERPs components related to selective attention tasks for the detection of schizophrenia (Neuhaus et al., 2011) and ERPs components acting as biomarkers of Substance Use Disorders treatment response (Houston and Schlienz, 2018). Another EEG approach is Event-related desynchronization/synchronization (ERD/ERS), consisting in non-phase-locked decreases/increases of EEG in frequency bands linked to specific sensory, motor and cognitive processes (Pfurtscheller, 1997. ERD and ERS can be observed in various brain sites at the same time, or in the same site at different times (e.g., ERD followed by an ERS; Pfurtscheller, 1997). ERD and ERS are particularly interesting in Brain Computer Interfaces (BCIs) and Affective Computing applications (Aftanas et al., 2001). Indeed, it has been found that the strength of ERD associated with physical motor execution (i.e., repetitive hand and grasping movements) might reflect time differences of hand coordination in the motor planning process or proprioception variation caused by hand movements (Nakayashiki et al., 2014). This process can be optimally applied in BCI systems that make use of motor activity or mental motor imagery. On the other hand, ERD/ERS method with enhanced density recording is well suited for elucidating the temporal and topographical structure of affective processing in different frequency bands, thus providing a source for objective emotional processing recognition (Aftanas et al., 2001).
	Delta waves δ—0.5/1 to 2/4 Hz	- Characteristic of deep sleep (especially during non-rem sleep phases N2 and N3) (Kandel et al., 2021), - Connected with health recovery, hypothalamic function (Chapin and Russell-Chapin (2013) and growth hormone release during sleep (Gronfier et al. (1996). - Predominant in EEG in infants (Demos (2005), decreases with age. (Clarke et al. (2001) - Alteration in the level of δ-waves during wake is detected in people with ADHD, learning disorders, and obsessive–compulsive disorder (Demos (2005); Sroubek et al. (2013) and is related to dementia (Soininen et al. (1982) and brain traumas (Gloor et al., 1977; Demos, 2005).	
	Theta waves θ—3/4 to 7/8 Hz	- Present in N1 and N2 non-rem sleep phases (Kandel et al., 2021). - Characterizes a state of drowsiness (Chapin and Russell-Chapin, 2013). - Linked to moments of creativity, meditation, imagery, recollection of past memories, hypnosis, or inattention (Klimesch, 1999; Demos, 2005). - Decreases with age (Clarke et al., 2001; Demos, 2005). - Scarcity of θ in the occipital lobe correlates with sleep disturbance, anxiety, and low resilience to stress or addiction (Chapin and Russell-Chapin, 2013). - Increase in people with ADHD (Demos, 2005).
	Alpha waves α—8 to 12/13 Hz	- Predominant when awake, typical of calm, idle, and focused relaxation. (Klimesch, 1999; Kandel et al., 2021) - Related to cognitive and intellectual performance. (Klimesch, 1999) - Increases with age (Clarke et al., 2001). - Declines or increases in relation to eyes open or closed (Demos, 2005). - Declines with age or reduced mental activity; can also be related to cognitive and memory impairment (Chapin and Russell-Chapin, 2013). - Abnormal activity can be linked to attention problems, ADHD, and sleep disorders (Sroubek et al., 2013). - Imbalance in α activity in the left frontal hemisphere is linked to depression (Demos, 2005). - Decrease of the lower alpha denotes attention, decrease in the upper alpha band is linked to semantic memory performance (Klimesch, 1999).
	Sensory Motor rhythm (SMR)—12/13 to 15/16 Hz	- Not a traditional division of wave bands, overlaps with high alpha and low beta. - Related to mental alertness, coupled with a still body (Chapin and Russell-Chapin, 2013). - Linked to attentional processing (Egner and Gruzelier, 2004).
	Beta waves β 13/16 to 21/40 Hz Low beta 13–21 Hz High beta 22–35 Hz	- Increased in adult age (Clarke et al., 2001). - Linked to relaxed focus and analysis, with an orientation toward the external (Demos, 2005). - Excessively protracted activation is registered in anxiety disorders, depression, obsessive–compulsive disorder, attention deficit disorder (Demos, 2005, chronic fatigue, and emotional volatility (Chapin and Russell-Chapin, 2013. - Excessive activity during sleep has been found in insomnia (Perlis et al., 2001). - Low β are related to focus, engagement, problem-solving (Chapin and Russell-Chapin, 2013), and attentional processing (Egner and Gruzelier, 2004). - High β correlate with concentration, high level of activation, attention, perception, cognition, and peak performance (Perlis et al., 2001; Demos, 2005), but also with anxiety and irritability and can relate to stress, anxiety, rumination, and obsessive thoughts, emotional intensity and hypervigilance (Demos, 2005); Chapin and Russell-Chapin, 2013).
	Gamma waves γ - 25/28 Hz to 42/100 Hz	- Related to cognitive efficiency in: learning, focus, insight, language comprehension, memory, (Chapin and Russell-Chapin, 2013) attention, perception, and cognition (Perlis et al., 2001; Fitzgibbon et al., 2004). - Scarcity is related to learning disorders and mental deficits (Demos, 2005), negative symptoms of schizophrenia, and Alzheimer’s (Herrmann and Demiralp, 2005), while abnormal activity is found in epilepsy and ADHD (Herrmann and Demiralp, 2005).

As shown in Table 1, biosignals are an especially important source of information for neurosciences because they are associated with specific correlates (such as brain activation states), which provide us with a series of implicit information that would not be possible to obtain using self-report tools such as questionnaires. These signals can be used in both offline and online applications. Offline applications include all clinical applications that deal with analyzing biosignals after they have been registered, and that allow researchers or medical teams to make inferences about an individual’s physical and mental state and, as a result, make decisions and diagnoses (Insel, 2014). Online applications, on the other hand, imply the real-time use of biosignals. In these applications, the biosignals serves as feedback about the individuals’ physical/mental state to allow them to visualize and eventually modify their own activations to achieve specific goals, as happens in BCI. It should be noted that even data relating to biosignals collected in a matter of minutes represent a massive amount of information that is extremely complex and difficult to analyze. For this reason, ML is increasingly used to extract information from these data to make it understandable and usable for medical and psychological sciences.

## 3. How to apply machine learning and AI to mental health and biosignals analysis

As previously stated, ML is data-oriented, which means that it requires a dataset containing quantifiable features as input (Shalev-Shwartz and Ben-David, 2014), often called “examples” (Goodfellow et al., 2016) since they serve as cases from which the algorithm infer relevant rules/patterns. Following that, depending on the model, examples of desired output may be required, to measure the correctness of the algorithm (Chollet, 2017).

The first fundamental step in applying ML to biosignals is data collection and dataset preparation. In fact, in order to reduce the computational cost (i.e., resources and time), and ease model interpretability, the dataset should include the minimum viable data, namely only the significant variables that have been selected to answer a specific research question.

After careful data collection, the pre-processing phase, which is intended to clean the data and make them readable, will include (Iniesta et al., 2016; Tuena et al., 2022):

- Cleaning: data needs to be screened to identify missing or incoherent elements, which should be corrected or discharged. It should be mentioned that each physiological data (e.g., EEG) needs a specific signal-cleaning process, to remove noise and artifacts. These errors, due for example to muscular activity or electromagnetic interference, could be identified manually or through automated statistical thresholding algorithms (Meisler et al., 2019).
- Data reduction: additional feature reduction may be implemented to reach a “minimum dataset.” Features could be aggregated or eliminated through specific algorithms if not found meaningful for the prediction.
- Data transformation: data might need to be scaled, decomposed, or aggregated. Physiological data are standardly analyzed in the time and frequency domains, which distinguish the spectral components in which raw data are usually transformed (Camm et al., 1996).

All the described processes are carried out in order to avoid the well-known AI drawback of *“garbage in, garbage out,”* which refers to the possibility of including errors or noise in the model if the input dataset has not been cleaned appropriately. If the training data is “garbage,” the model will be trained and validated on non-representative or insufficient information, resulting in either a lack of generalizability or an incorrect (or biased) way of making predictions on new data. It must be emphasized that biases are often embodied in the human logic with which examples are chosen; an example of this phenomenon is the racial bias that might lead to the inclusion in the input dataset of information solely related to white people (Ledford, 2019; Obermeyer et al., 2019). If the model is intended to be used in other types of ethnicities, this bias will result in less generalizability and fairness. Therefore, a model can be considered effective not only if it achieves high levels of performance and accuracy, but also if the predictions it makes are inclusive and representative of various types of case studies.

### 3.1. Training

Without explicit instructions, ML seeks to identify broad principles underlying a set of data through a process characterized by few formal assumptions (Bzdok and Meyer-Lindenberg, 2018). ML algorithms are “model agnostic”: their structure and design are independent of the problem that they are going to face, and are chosen based on the empirical performance among general-purpose learning algorithms (Orrù et al., 2019).

**Figure 1:**
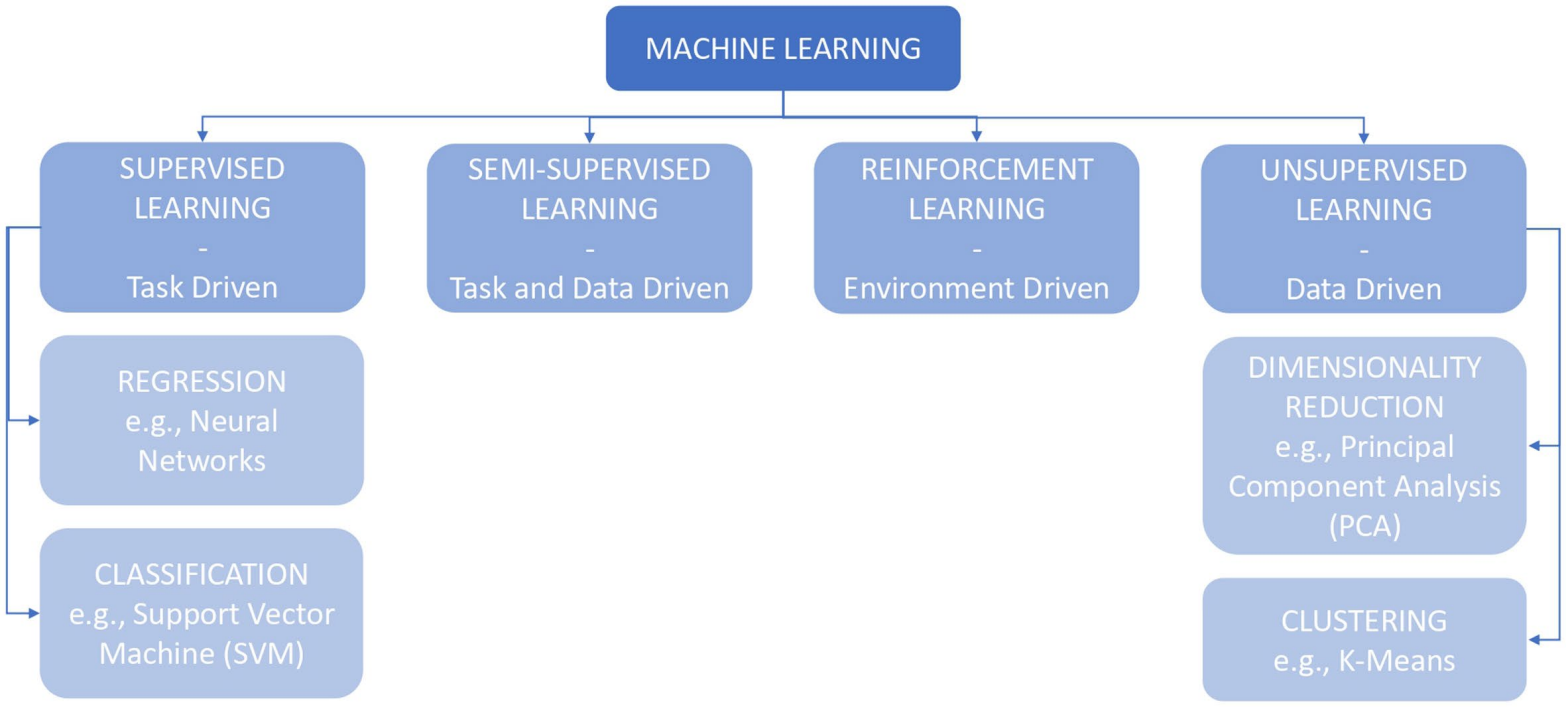
Machine learning (ML) family and respective different types of learnings.

As shown in Figure 1, different types of ML training can be implemented by the researchers, depending on their objectives and on the information on data available/provided to the algorithms (Goodfellow et al., 2016).

The term *“supervised learning”* refers to the idea of having someone instructing the machine about the characteristics of the data, on which the learning process is dependent: the input dataset should contain examples paired with labels or targets, which describe significant features of the linked data (Goodfellow et al., 2016; Chollet, 2017; Choi et al., 2020). The algorithms following this learning principle are *classifiers* and *regressors*, which differ in terms of the output variable: classification algorithms’ outcomes are categories or classes, while regression algorithms predict continuous variables or probabilities (Shalev-Shwartz and Ben-David, 2014; Goodfellow et al., 2016; Orrù et al., 2019; James et al., 2021).

A similar kind of learning process is *semi-supervised learning*, in which the algorithms are trained on both labeled and unlabeled data (Chollet, 2017; Choi et al., 2020).

Conversely, in *unsupervised learning*, the data used to train the algorithms are completely unlabeled and there is no difference between the training and the test sets: the machine is left unsupervised in discovering patterns from the examples. The researcher is unaware of, or chooses not to disclose, additional information about the data: this approach may be advantageous in the exploratory phase of research, for example, when looking for similarities in physiological activation patterns during different behaviors (Goodfellow et al., 2016; Chollet, 2017; Choi et al., 2020).

Lastly, in *reinforcement learning*, the algorithm is required to obtain a specific result (e.g., winning a game of Go), without receiving constraints on the choice of the actions which lead to the target. This model is mostly applied in games (e.g., DeepMind), currently lacking possible applications in other fields, such as clinical medicine (Chollet, 2017; Choi et al., 2020).

*Supervised models*, wherein examples provided to the algorithm contain a label, are widely applicable to healthcare contexts, and clinical research and practice (Jiang et al., 2017; Tuena et al., 2022). Jiang et al. (2017) reviewed the ML algorithms applied in healthcare for imaging, genetics, and electrophysiological data: Support Vector Machine (SVM; 31%) and Neural Networks (NNs; 42%) are the most common (Jiang et al., 2017).

*Support Vector Machine (SVM)* is used to divide subjects into two categories (Jiang et al., 2017); it classifies data by creating a linear or hyperplanar boundary, between them, then computes only the data closest to the border of the resulting distributions (the support vectors). The margins of the boundary and the degrees of misclassification can be balanced to reach a correct classification in the training set in order to correctly apply the algorithm to new unlabeled data (Chollet, 2017; Dwyer et al., 2018).

*Neural Networks* (NNs), or *artificial NNs*, are biologically inspired models structured in nodes (mimicking neurons’ bodies) and connections (mimicking axons and dendrites). This algorithm is able to discover complex non-linear relationships between input and the desired output data through multiple hidden layers (non-interpretable), modifying the weights of the connections among these layers to obtain the correct class prediction (Shalev-Shwartz and Ben-David, 2014; Jiang et al., 2017; Orrù et al., 2019; Choi et al., 2020).

*Decision Trees* algorithms are notable for their less complex interpretation and visualization, as they design a tree-shaped decision flow with if-then rules splitting the data at each node based on the thresholding value. Although some models are computationally expensive, these algorithms are particularly interpretable and can be a useful tool in neuroscience and biosignals processing for mental health. Random Forest and Gradient Boosting Machines can be thought of as extensions of decision trees (Shalev-Shwartz and Ben-David, 2014; Chollet, 2017; Orrù et al., 2019; Choi et al., 2020).

Among the *unsupervised learning* methods, clustering and Principal Component Analysis (PCA) are the most used in health, and in physiological patterns analysis (Jiang et al., 2017).

*Clustering* consists in representing unlabeled data with similar features partitioned in multiple groups (clusters). Examples of clustering are k-Means clustering, in which data are divided into k non-overlapping groups starting with k different centroids (Goodfellow et al., 2016; Bzdok and Meyer-Lindenberg, 2018), and hierarchical clustering, in which data are modeled on a nested tree, through successive fine-grained non-overlapping divisions (Bzdok and Meyer-Lindenberg, 2018).

*Principal Component Analysis (PCA)* is used to reduce the number of dimensions, discarding less informative features, especially when the variables have a large number of dimensions. Data is projected in a few Principal Component (PC) directions, represented as vectors. PCA is applied for example in both genome studies and EEG recordings. Interestingly, different methodologies can be combined to extract more information from the data. PCA can be used to reduce the dimensions of the data (e.g., multimodal physiological data recordings from different biosensors), and then clustering can be used to group the resulting data (e.g., clustering subjects, patterns of activations, epochs with similar physiological correlates, etc.; Goodfellow et al., 2016; Huys et al., 2016; Jiang et al., 2017).

### 3.2. Validation and test

**Figure 2:**
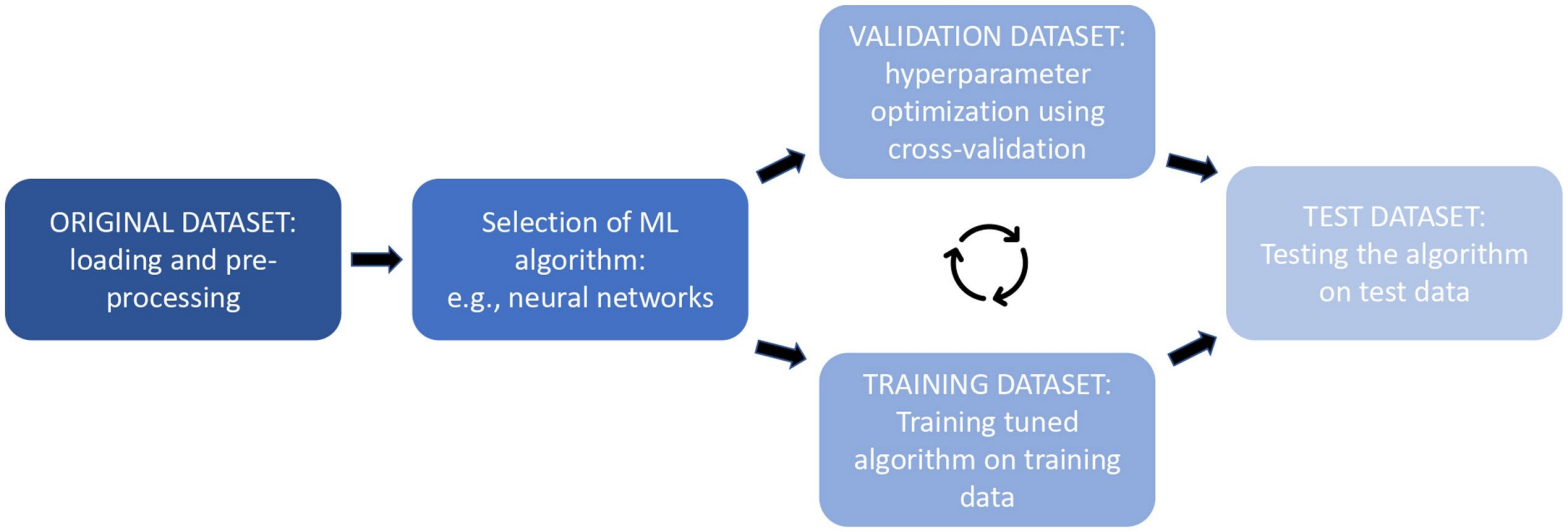
Representation of the training, validation, and testing processes in order.

The logic behind both validation and test is one of the main points which could help behavioral and neuroscientific research to overcome criticism on replicability and reproducibility and improve the generalization of results (Bzdok and Meyer-Lindenberg, 2018; Orrù et al., 2019). Generalization is indeed one of the main aims of ML, but when ML models are trained repeatedly on the same data, they result in an excessive adaptation to the examples provided (a phenomenon called “overfitting”), losing reliability on new datasets, thus disproving the efficiency of the algorithm (Kuhn and Johnson, 2013; Chollet, 2017). To avoid this problem, the initial data need to be divided into different datasets, one for the training, one for validation, and one for the test (Figure 2).

From the original dataset is firstly extracted the test dataset; after that validation is performed through the creation of a further *“hold-out validation set”*: a bigger training dataset is used to proceed with the training, while a smaller *held-out* validation set is used to perform the evaluation of the learning. But since the held-out data could not be totally representative, the K-fold cross-validation approach is considered more accurate and is particularly effective when a large amount of training data is available. The initial data are divided into number K partitions (usually 10), then the training includes the data of all-minus-one partitions and the process is repeated with the remaining K–1 partitions. Each repetition is called a fold; the final validation score will be the average of all the fold validations (Kuhn and Johnson, 2013; Shalev-Shwartz and Ben-David, 2014; Goodfellow et al., 2016; Chollet, 2017). Leave-one-out cross-validation is a subtype of k-fold validation that is used when the numbers of instances are equal to k, usually happening when a small dataset or small numerosity of a certain class is present (Wong, 2015). This method is commonly used in biosignal registration studies when the stream of data (i.e., the psychophysiological recording) from each subject is large, but the subject’s numerosity is scarce: the validation, therefore, leaves out an entire subject registration each fold (Picard et al., 2001; Koelstra et al., 2012; Soleymani et al., 2012).

The test phase would also benefit from greater data availability: following the selection of the best model, the performance of the output predictor is assessed on the third set of data, kept aside before the learning phase, also known as the “test set” (Shalev-Shwartz and Ben-David, 2014) or “holdout test set” [suggested size is around 20% of the initial dataset (Orrù et al., 2019)]. The computed value is used as an estimate of the trained predictor’s real error of the model (Shalev-Shwartz and Ben-David, 2014), providing a percentage of its accuracy, sensitivity, and specificity of its performance, or of its generalization error (Goodfellow et al., 2016; Dwyer et al., 2018). If the test phase fails, the researcher could intervene by enlarging the dataset, modifying the hypothesis, changing which features of the data were considered more representative, or modifying the chosen algorithms (Shalev-Shwartz and Ben-David, 2014).

After the testing phase, to better evaluate the generalization capacity of the realized model, especially in the health sector, a substantial, representative, and a more current sample of data should be used to further test it in the actual world (Bzdok and Meyer-Lindenberg, 2018).

## 4. What AI, biosignals, and machine learning can do for mental health

Some actual applications of biosignals and ML in the mental health field are now provided, trying to showcase the potential and the various areas in which these technologies could be applied. The research articles reviewed in this section are summarized, in Table 2 (Diagnostic Precision Medicine), Table 3 (Affective Computing), and Table 4 (BCI).

**Table 2:** The milestones research studies in Diagnostic Precision Medicine.

Article and year	Topic	Experimental paradigm	No. of subjects	Biosignals	Feature selection modality	Classification	Validation	Accuracy
Chabot et al. (1996)	Discrimination between ADD or ADHD, and SDLD in children	30 min of rested eyes-closed EEG	407 ADD/ADHD, 127 SDLD, and 310 control children	30 min of rested eyes-closed EEG	Not reported	Discriminant analyses (three-way discriminant and two-way discriminant)	Not reported	3-way 88.7% ADD/ADHD, 69% SDLD, and 76.1% control. 2-way 93.1% ADD/ADHD (97.0% new data), and 89.7% for SDLD (84.2% new data). The response to amphetamines in ADHD 75.6–75.8%
Mueller et al. (2010).	Discriminate adult ADHD subjects from healthy controls	74 ADHD, 74 controls	GO/NOGO paradigm	EEG and ERPs	Extraction from the independent components	Non-linear SVM	10-fold cross-validation	92% correct discrimination between ADHD and controls
Tzimourta et al. (2018)	Seizure detection and prediction	Database’s analysis (recordings subdivided by ictal, preictal, and interictal, and into 2-s epochs)	21 patients’ EEG data extracted from the database of the EPILEPSIAE project.	EEG	energy, entropy, standard deviation, variance and mean of the absolute values of the wavelet coefficients	SVM	10-fold cross-validation	93.78–100%
Regalia et al. (2019).	Detect generalized tonic–clonic seizures with wearable wristbands	Data retrieved from the E4 were paired to the more invasive video-electro-encephalography (v-EEG),	9–18—135 epilepsy patients	Accelerometers and SC + video-electro-encephalography (v-EEG) as gold standard	Not reported	Supervised learning method	Not reported	92–100%

**Table 3:** The milestones research studies in Affective Computing.

Article and year	Topic	Experimental Paradigm	N. of subj	Biosignals	Feature selection modality	Classification	Validation	Accuracy	More relevant features
Picard et al. (2001)	Discrimination among anger, hate, grief, platonic love, romantic love, joy, reverence, and no emotion	Daily 25-min recording for 30 days, recalling important images that were individually linked to the states	1	Facial EMG, blood volume pressure, SC, and respiration	Hybrid Sequential Floating Forward Search with Fischer Projection	k-nearest-neighbor (k-NN) and a Maximum *a Posteriori*	Leave-one-out cross-validation	81% for the emotional profiles (best results for anger, grief, joy, reverence, and no emotion)	HR, SC, and respiration are discovered in all of the most accurate outcomes
Kim and André (2008)	Detection of joy, pleasure, sadness, and anger, discriminated for high/low arousal and valence	Recordings listening for personally selected songs, linked to the target states	3	EMG, ECG, SC, and respiration	Sequential backward search	Linear discriminant analysis	Leave-one-out cross-validation	95% in-subject and 70% intra-subject	EMG and SC for arousal, ECG and respiration for valence
Koelstra et al. (2012)	Detection of arousal and valence levels	Recordings watching 40 musical videos, then rated for arousal, valence, and dominance	32	EEG, SC, respiration, skin temperature, ECG, BVP, Zygomaticus and Trapezius EMG, and EOG	Fisher’s linear discriminant	Naive Bayes	Leave-one-out cross-validation	65% for features extracted from the videos, 62% for EEG, and 57% for peripheral physiological signals	Features extracted from the videos
Soleymani et al. (2012)	Detection of arousal and valence levels	Recordings watching 20 videos, then evaluated by valence and arousal	24	EEG, ECG, SC, respiration, skin temperature, and eye glaze	ANOVA test	Support vector machine with a radial basis function kernel	Leave-one-out cross-validation	68.5% for valence and 76.4% for arousal (3 levels)	EEG features: gamma for valence; slow-alpha, alpha, and theta for arousal
Zheng and Bao-Liang (2015)	Discrimination of positive, neutral, and negative emotions	Recordings watching 15 movie clips linked to positive, negative, and neutral emotions	15	EEG and EOG	Performed automatically during the classification	Deep Neural Networks	Not reported	86.08%	Increase of beta and gamma for positive emotions; lowering in beta and gamma for neutral and negative emotions; increase in alpha for neutral emotions.

**Table 4:** The milestones research studies in Brain-Computer Interfaces.

Article and year	Biosignals	Brain input	Task	Objective
Blankertz et al. (2007) Hex-O-Spell	Sensorimotor rhythms	Two different imagined movements (e.g., right hand and right foot movements)	Rotation of an arrow pointing to 6 hexagons	Communication
Krepki et al. (2007) The BBCI framework	Eeg	Multiple trials of right and left-hand movements (imagined and executed)	Commands selection (left/right/rest)	Communication/interface with the environment
Leeb et al. (2007)	Beta oscillation	Imagined movement of feet	Movement of an avatar	Navigation in a VR space
Pfurtscheller et al. (2000) The Graz-BCI	Eeg	Motor imagery (e.g., right-hand versus left-hand movements).	Moving on an horizontal bar to the right or left	Different control option in one dimension (e.g., remote control interacting with the environment)
Rebsamen et al. (2010)	P300	Reactive BCI	Selection of a destination among pre-definite locations in a VR house	Wheelchair movement
Muller-Putz and Pfurtscheller (2008)	SSVEPs	Reactive BCI	Discriminate four classes of commands (left, right, open, and close)	Motion of a two-axes- hand prosthesis
Gao et al. (2003)	SSVEPs	Reactive BCI	Selection on a 6×8 visual panel, connected to an infrared remote controller	Use of a remote controller, with 48 target functions for interfacing with household appliances
Vaughan et al. (2006) Wadsworth BCI	Sensorimotor rhythms and P300	Motor imagery and Reactive BCI	Movement of a cursor in two dimensions and item selection in a 6×6 matrix (letters and symbols)	Interface device for a domestic environment
Li et al. (2010)	P300 and Mu/Beta rhythm	Reactive BCI and motor imagery	2D cursor movement (horizontal and vertical)	Interaction

### 4.1. Diagnostic precision medicine

Differential diagnosis through *Precision Medicine* arose in a predominant way in oncology, where the need for detailed clinical subtyping is fundamental for adequate targeted drug treatment (Gibbs et al., 2018). Even if broad therapeutics have been shown to be beneficial in Neurological and Neuropsychiatric Disorders, the ability to target narrow groups of patients, likened by similar underlying genetic variations or with the same subtype of pathology, may be preferable. But so far, traditional Psychiatry refers to the ICD-10 and the DSM-5, thus grounding its diagnoses primarily on a list of symptoms. Further studies and research are still needed to discover reliable biomarkers, which can contribute to the formulation of more precise and objective diagnoses (Insel, 2014). In a new era of evidence-based psychiatry, objectively measurable endophenotypes may allow for earlier disease detection, more personalized treatment selection, and dosage adjustments (Bzdok and Meyer-Lindenberg, 2018). Machine Learning, which can handle large multimodal databases and Big Data, may enable in the transition from a treatment targeting a heterogeneous clinical population to a stratified clinical group with a common disease (Gibbs et al., 2018) or even focus prediction on a single subject level (Cipresso and Immekus, 2017; Orrù et al., 2019; Rutledge et al., 2019; Tuena et al., 2020).

Chabot et al. (1996) explored how to discriminate between children with Attention Deficit Disorder (ADD) and Attention Deficit Hyperactivity Disorder (ADHD) and children with Specific Developmental Learning Disorders (SDLD). 30 min of rested eyes-closed EEG was recorded for 407 ADD/ADHD children, 127 SDLD children, and 310 children without specific health problems and used as a control group. EEG signals were cleaned, converted with the Fast Fourier Transform, and Quantitative EEG (QEEG) features were calculated. Discriminant analyses and a split-half of the relevant populations were performed to classify the three groups (three-way discriminant), SDLD from ADHD/ADD (two-way discriminant), and two groups of ADHD children, responding to two different amphetamines. The obtained accuracy in the three-way discriminant identified 88.7% of ADD/ADHD, 69% of SDLD, and 76.1% of control subjects. The two-way discriminant obtained a 93.1% accuracy for ADD/ADHD, and 89.7% for SDLD, while fresh data were classified with, respectively, 97.0 and 84.2% accuracy. The response to amphetamines in ADHD was predicted between 75.6 and 75.8% (Chabot et al., 1996). This study demonstrates how ML can be used to improve clinical practice, trying to reach the ideal concept of Precision Medicine.

In more recent years, works on ML classification of mental disorders or neurodiversity emerged as a novel research trend to assist mental health professionals. Mueller et al. (2010) develop another framework to discriminate ADHD subjects from healthy controls in the adult population. Two groups balanced for age, sex, and number (74 ADHD, 74 controls) underwent a GO/NOGO paradigm, while their EEG was acquired. Event-related potentials (ERPs) were calculated, and through an independent component analysis, ERPs were decomposed into independent components. Feature selection was performed by extracting them from the independent components, and a non-linear SVM was implemented for the classification phase, with 10-fold cross-validation. The results showed an accuracy of 92% for the correct discrimination between ADHD individuals and controls (Mueller et al., 2010). Buitelaar et al. (2022) reviewed the diverse kinds of biomarkers that precision medicine can use to diagnose ADHD or predict the response to stimulants. EEG or QEEG are widely reported, but just three studies addressed the problem through an experimental design based on ML techniques and they were all concerning fMRI or MRI data (Buitelaar et al., 2022).

Machine language and QEEG are also applied to major depression for predicting the best response to antidepressants. The Psychiatric EEG Evaluation Registry (PEER) is a database with patients’ QEEG recordings, both drug-free and under pharmacological treatment (Iosifescu et al., 2016; Schiller, 2018). PEER Interactive interconnects with the database elaborating a prevision through two ML classifiers, one structured on “net hints” and the other on a logistical regression (Schiller, 2018). Iosifescu et al. (2016) evaluated 2 years of PEER Interactive use with a randomized double-blind design on patients with depressive disorder diagnosis, finding a significant reduction in suicidal ideation and improvements in both depression and post-traumatic stress disorder (PTSD) scores (Iosifescu et al., 2016).

In epilepsy, seizures are diagnosed and monitored using neuroimaging and electrophysiology. EEG is used by Tzimourta et al. (2018) to develop computerized methods for automating seizure detection and prediction. 21 patients’ EEG data were extracted from the database of the EPILEPSIAE project. Recordings were subdivided by ictal, preictal, and interictal (the moments during, preceding a seizure, and the lapse between seizures), and cut into 2-s epochs. The signals were then decomposed by the Discrete Wavelet Transform and features were calculated. A SVM paired with 10-fold cross-validation was then structured to classify the interictal moments from seizure-free periods. Accuracy ranged between 93.78 and 100%, while sensitivity was between 45.30 and 100% (Tzimourta et al., 2018).

Ulate-Campos et al. (2016) describe which automated seizure detection systems can be found in literature: EEG, surface EMG, SC, ECG, skin temperature, and respiration are reported as viable instruments, but only technologies based on EEG and ECG are described applying predictive algorithms. In addition, are noteworthy the reported multi-modal system which integrates different data sources using SVM algorithms. In the Multi-modal intelligent seizure acquisition (MISA) system, the information from the body movement is blended with EMG data and used to predict motor seizures (Conradsen et al., 2012), while Shoeb et al. (2009) detect seizures by integrating EEG and ECG signals (Shoeb et al., 2009; Ulate-Campos et al., 2016).

As the last point, wearable devices are becoming important allies in bridging lab studies with more ecological investigations: a special interest is devoted to chest bands and wristbands able to detect ECG data, and in some cases, SC, due to their low price and the possibility of being worn comfortably for a long time, during the everyday life. Some studies also focus on EEG wearable devices, even if their usage in an ecological environment appears more complex.

About this topic, Perna et al. (2018) describe how wearable biosensors and the analysis of electronic medical records can access massive quantities of data and become important instruments for applying precision medicine to psychiatry, especially with the support of supervised and unsupervised ML models (Perna et al., 2018). Welch et al. (2022) work is instead focused on how ML enables to assess of psychiatric disorders in young people through wearable devices. The review finds ECG and EEG data (SC was additionally considered in the experiments involving Empatica E4) alongside information about movement, are considered in studies aiming to individualize diagnosis and the treatment of several psychiatric disorders (i.e., ADHD, learning disability, and autism spectrum, bipolar, and internalizing disorders; Welch et al., 2022).

An exemplifying application of a wearable device to epilepsy can be found in Regalia et al. (2019): a ML model was designed to determine the effectiveness of the combined use of accelerometers and SC sensors to detect generalized tonic–clonic seizures, relying on the E4 wristbands (Empatica) for the detection of motion and SC. Data retrieved from the E4 were paired to the more invasive video-electro-encephalography (v-EEG), the gold standard for seizure detection, and labeled consequently. A supervised learning method was then trained to predict generalized tonic–clonic seizures. Learning results offer an elevated sensitivity (92–100%) in seizure detection, and lower false alarms, better differentiating seizures from real-life activities which could lead to similar movements (e.g., clapping hands, brushing teeth). In addition, further quantification of the seizure-induced autonomic dysfunction was made possible (Regalia et al., 2019).

These studies demonstrate how ML can be used to improve precision medicine and clinical practice. EEG and QEEG pattern evaluation through ML seems a promising field for Mental Health, offering great opportunities for accelerating the diagnostic process and getting it out of the binary schemes of lists of symptoms. Despite this, accuracy percentages do not reach sufficient levels for a diagnostic process: further research is needed to apply the described procedures to standard clinical approaches.

### 4.2. Affective computing

One of the main topics in psychology involves emotions on all their sides, from theorization to treatment: relations between emotions and physiological modifications have been studied since, at least, the end of the 19th century (Cacioppo et al., 2000; Bradley and Lang, 2007) and already in 1958 John Lacey correlated variations in biosignals to emotional states (Cacioppo et al., 2000; Bradley and Lang, 2007). Effective categorization of emotive reactions to stimuli is also found in their distribution in a cartesian space created around two perpendicular axes, as in Russel’s Circumplex Model of Affect (pleasantness and activation; Russell, 1980) or in Lang’s organization on Affective Valence and Arousal (Lang, 1995).

Despite strong theoretical bases linking emotion to physiological reactions, a lack of consistency is found in delineating replicable or generalizable patterns arising from autonomic activity, possibly due to issues in transporting theory to methodologically reliable experiments (e.g., emotion induction, self-report observations; Cacioppo et al., 2000; Bradley and Lang, 2007).

New possibilities of development and overcoming the complexities related to the elaboration of emotional patterns arise from the interest that the world of computer science has begun to turn to these themes: researchers start to theorize that the aim of the implementation of a strong AI would not be accomplished without considering an emotional part (Barrett et al., 2016). Specifically, Picard (2000, 2003) theorizes how the ability to recognize human emotion will be needed by computers in the near future, so that they can be useful to humans in the best way (e.g., user interfaces): Affective Computing is defined as *“computing that relates to, arises from, or deliberately influences emotions”* (Picard, 2000) and one of its main challenges relies on using AI to model an Affective System able to recognize emotion from humans, e.g., from expressions, gestures, vocal intonation and, especially, biosignals. This approach will lead to improvement in human–computer interaction (HCI; Picard, 2000) and to a further understanding of human emotions.

**Figure 3:**
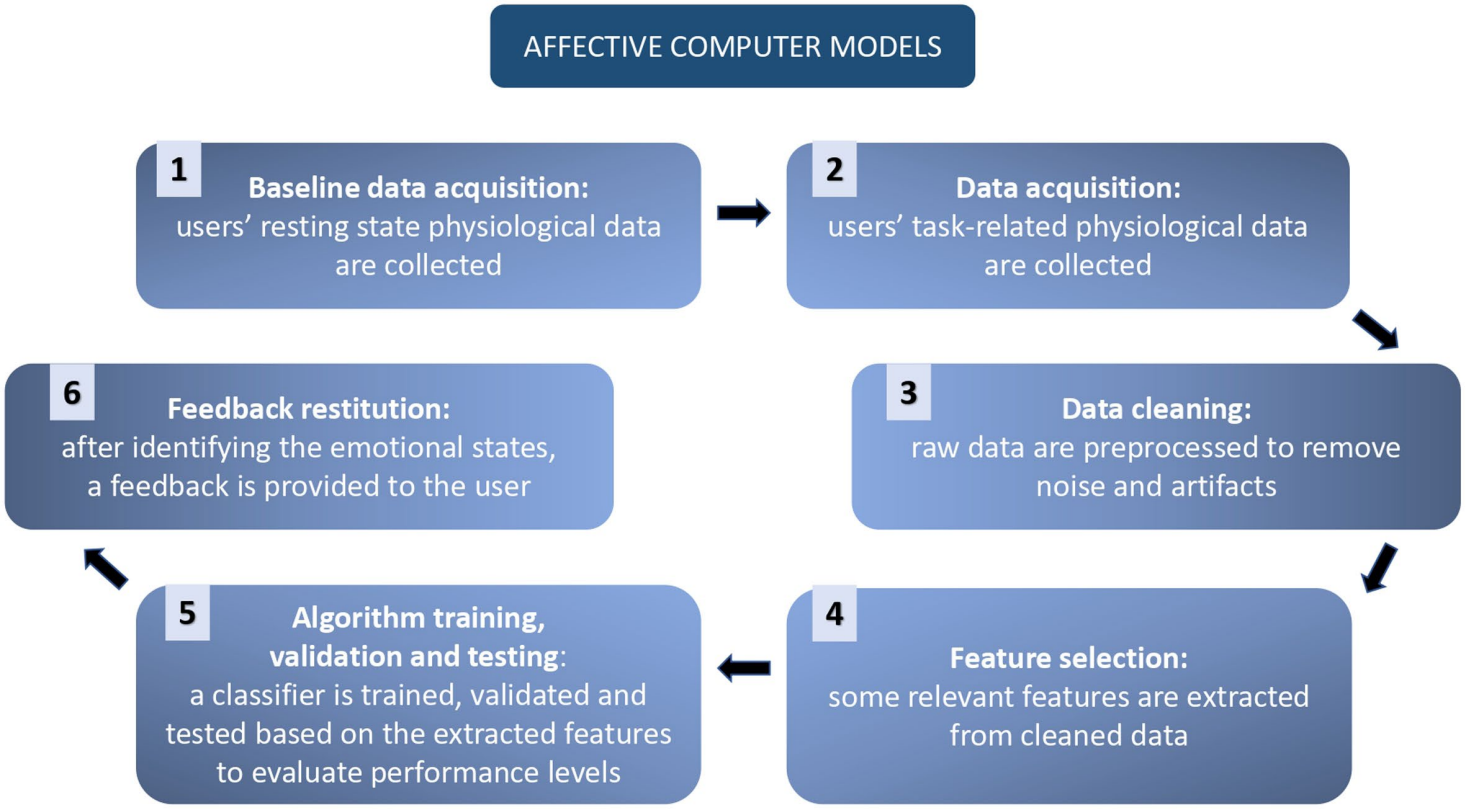
Representation of Affective Computing workflow.

Affective Computer models typically follow this standard workflow (Zheng and Bao-Liang, 2015; Figure 3). The first passage is represented by physiological data acquisition. Subjects are connected to various devices, which can record multiple physiological activations. Then the “ground truth” is recorded: some minutes of the person’s activation at a resting state are collected to be used as a baseline. This level of activity is often subtracted from the stream of data during the experimental phases, to decrease the influence of interpersonal differences.

Then the subject is asked to perform some tasks or is presented with different stimuli, which should elicit different levels of arousal and valence, or different emotions: this will permit the labeling of the data.

Physiological data are successively cleaned from errors and noise, and sub features of the signals are calculated (e.g., EEG bands, HRV), and inserted in a database with the proper label. From the algorithmic point of view, the learning phases consist of the following steps:

- Feature selection, reduction, or aggregation is performed through supervised and unsupervised learning models. This phase aims to reduce the computational cost of the subsequent parts.
- Supervised learning models are run to obtain the classification of the different emotional patterns. In the recent literature the most frequently applied classifier is SVM, followed by k-Nearest Neighbor, Random Forest, Linear, Quadratic Discriminant Analysis, Neural Networks, and Decision Tree. Unsupervised learning models, especially Deep Learning, are reportedly gaining popularity since they allow to skip the feature selection phases (Bota et al., 2019).
- Validation, *via* leave-one-out cross-validation.
- Test phase, in which the levels of performance are evaluated.

At the end, a feedback system can be addressed to the user (Zheng and Bao-Liang, 2015).

In one of the first and most cited affective computer articles, Picard et al. (2001) describe a system for the discrimination of multiple emotions. One subject underwent a daily 25-min registration for 30 days, in which she recalled meaningful imageries, individually connected to anger, hate, grief, platonic love, romantic love, joy, reverence, and no emotion. Meanwhile, her physiological reactions were collected, *via* facial electromyography, blood volume pressure, SC, and respiration.

Feature selection of data was performed by creating a Hybrid Sequential Floating Forward Search with Fischer Projection, a novel combination of Sequential Floating Forward Search, which selects features directly, and Fischer Projection (FP), which returns a linear transformation of features. The learning phase involved a k-Nearest-Neighbor (k-NN) classifier and a Maximum *a Posteriori* classification, with leave-one-out cross-validation. Results showed 81% of accuracy in the discrimination of the eight emotional profiles, with the best results for anger, grief, joy, reverence, and no emotion. The most robust features are found to play a role in all the most performative results and are linked to HR, SC, and respiration (Picard et al., 2001).

In Kim and André (2008) the emotions of joy, pleasure, sadness, and anger are also discriminated for their arousal and valence levels (e.g., joy: high arousal and high valence; sadness: low arousal and low valence). Three subjects were asked to select four songs, which could induce sensations related to the target emotions. Their EMG, ECG, SC, and respiration activities were then recorded, while they were listening to the selected music. Successively, the sequential backward search method was applied to perform the feature selection. An extended linear discriminant analysis (pLDA) was chosen for the classification learning, demonstrating better performance than k-NN, and multilayer perceptron. The direct multiclass classification was furthermore confronted with a scheme for decomposing it in a structure of two-class problems: the emotion-specific multilevel dichotomous classification. In this framework, the emotional patterns were grouped and confronted both per arousal and per valence reaching 95% of accuracy in-subject and 70% intra-subject. Analyzing the features, a correlation between arousal and both EMG and SC was found, and connections between valence levels and ECG and respiration features (Kim and André, 2008).

Also, Koelstra et al. (2012) chose the evaluation for valence and arousal to discriminate emotions, adding also dominance and familiarity. The study resulted in the construction of the open-source DEAP database, in which EEG, SC, respiration, skin temperature, ECG, BVP, Zygomaticus and Trapezius EMG, and electrooculogram (EOG) of 32 subjects were collected. From a bunch of 120 musical videos, previously rated by valence, arousal, and dominance, the 40 with stronger ratings were selected. The videos were then presented to the subjects, and after that, they were asked to rate them by arousal, valence, dominance, and liking. Features selection was performed through a Fisher’s linear discriminant for EEG, the whole set of peripheral physiological signals, and some features extracted from the videos. Successively, a single-trial structure classification aimed to binary-discriminate the three groups of data, in terms of levels of arousal, valence, and liking. A Naive Bayes was chosen as a classifier due to its ability to cope with unbalanced classes, as the result of an unbalanced subjects’ rating of the videos. The leave-one-subject-out cross-validation and an F1 measure were applied to improve and evaluate the performance. Results showed that the best classification was obtained with the features extracted from the videos (65%), while no significant difference was found between EEG (62%) and peripheral physiological signals (57%) performances (Koelstra et al., 2012).

Soleymani et al. (2012) proposed a similar structure, aiming to discriminate the affective connotation of selected video clips. 20 videos, previously rated for arousal and valence, were shown to 24 subjects, while their EEG, ECG, SC, respiration, skin temperature, and eye glaze were recorded. Following each clip, participants evaluated them by valence and arousal. Data preprocessing for noise removal was then performed, features from EEG, eye blinking, and gaze distance were calculated, while through a principal component analysis was estimated the pupil diameter changes. A further feature selection *via* ANOVA test was performed for each cross-validation. The classification phase was performed thanks to a support vector machine with a radial basis function kernel and validated through a leave-one-participant-out cross-validation. Additional fusion strategies were applied both to the feature selection and in the decision phase. Results showed the best accuracy of 68.5% for three levels of valence and 76.4% for three levels of arousal, both obtained thanks to decision fusion. The EEG features better contributing to the classification were linked to slow-alpha, alpha, and theta for arousal, and to beta, and gamma for valence (Soleymani et al., 2012).

Zheng and Bao-Liang (2015) explore the opportunities opened by Deep Neural Networks (DNNs) in the analysis of EEG patterns during different emotions. Fifteen movie clips linked to positive, negative, and neutral emotions were submitted to 15 subjects, while their EEG signals and EOG were registered. After each movie session, an assessment of their feeling, their knowledge, and their understanding of the film were collected. DNNs approach permits skipping the classical feature selection process, selecting features automatically during the classification phase: just differential entropy was extracted for each EEG band and for every 62 EEG channels. Differential asymmetry and rational asymmetry features were added to investigate the lateralization of brain activity, calculating rational asymmetry differences and ratios of couples of electrodes. The classification was performed using the DNNs model Deep Belief Network, whose training passes through two unsupervised and one supervised phase. The final results showed a connection between positive emotion and an increase of beta and gamma bands, while neutral and negative emotions lead to a lowering in beta and gamma activation. Neutral emotions showed an increase in alpha instead. The DBN performance reached 86.08% accuracy in differential entropy classification. As the last step was performed an “Electrode Reduction,” choosing a set of 4, 6, 9, and 12 channels: the performance even increased to 86.65% for the 12 channels solution, and remained quite stable also for the solutions with few electrodes, with a result of 82.88, 85.03, and 84.02%, respectively, (Zheng and Bao-Liang, 2015).

New frameworks are embedding Affective Computing in more complex systems, in which the detected emotion can be reported to the users, in order to let them modulate their behavior, following the typical pattern of Biofeedback and Neurofeedback interventions. Multiple studies evaluate complex emotions in real-life scenarios and open the possibility of a prospective human-computer interaction system, which can offer useful feedback to the users.

For example, during a real-world driving task, Healey and Picard (2005) reveal how to collect and analyze physiological data to ascertain the relative level of stress experienced by the driver. 24 subjects’ ECG, EMG, SC, and respiration were recorded during at least 50-min sessions of driving. The route was designed to possibly pass-through situations that could lead to different levels of stress. Subjects completed questionnaires to assess their level of stress related to specific driving events, and furthermore, an evaluation of the events was performed by the experimenters. These data were merged to obtain a continuous stress metric. Analysis was performed considering two-time frames, 5 min, and 1 s. In the first case, a FP matrix and a linear discriminant were applied in the feature selection phase. Then a classification was performed, and stress was detected with a 97.4% accuracy. In the second case, was calculated the correlation coefficients between the stress metric and the features derived from the physiological signals; HR and SC offered the highest correlation (Healey and Picard, 2005).

AffectAura described in McDuff et al. (2012), instead offers a continuous multimodal system of affect recognition paired with a user interface to visualize it. The proposed objective aims at stimulating users to an introspective reflection on their “affect timeline,” illustrated by valence, arousal, and engagement. To obtain the affect recognition, a ML system was trained to predict levels of valence, arousal, and engagement (low, high) from multimodal data (Webcam, microphone, Kinect, SC sensor, and File Activity) of five subjects, who needed to assess their state at standard intervals of time. A Nearest-Neighbor classifier was applied for the first learning supervised phase; a Neighborhood Component Analysis was then used to match new data to the most similar annotated data already in the system. A validation leave-one-out was performed and lead to a 68% of global performance. A usability study was then conducted, in which users were successfully helped to reconstruct emotional events of the week and most of them found AffectAura “useful for reflecting on events collected” (McDuff et al., 2012).

The examples listed here represent milestones in the Affective Computing research line. This topic is, in recent years, sparking profound interest both in the world of neuroscience and psychology, and in that of computer science and engineering, but often separately: a meeting point with a common language, techniques, and standards should be further stimulated, aiming at a greater union of forces, knowledge and approaches.

Many works are portrayed in recent literature illustrating the evolution of Affective Computing to date, and some of its possible applications. For example, Bota et al. (2019) in addition to the most commonly used biosignals and the ML techniques for features selection and classification, describe the additional experimental phases and materials essential to the construction of an emotion recognition study. They list the most common emotion elicitation materials, such as various kinds of media (e.g., pictures, videos, music) to be shown to the subjects, and describe controlled settings, to create a reproducible environment. Assessment methods, with which the subjects annotate their elicited state, are described thereafter. The most reported instruments are the Self-Assessment Manikins (SAM), the Ecological Momentary Assessment/Experience Sampling Method, and the PANAS questionnaire (Bota et al., 2019).

Several studies dedicate a special focus to EEG, such as Al-Nafjan et al. (2017), who describe the innovations in EEG emotion recognition systems. Among the studies reviewed, attention is given to those which apply ML: the reported classification algorithms are SVM, k-NN, Linear Discriminant Analysis, and Artificial NN. The feature extraction phase has gained specific attention because it is described as one of the principal difficulties in the EEG emotion recognition system. More in detail, the researchers summarized that, alongside the classical statistical features, in the frequency domain the power spectral density (i.e., band powers) is obtained through the Fourier Transform; in the time domain, Hjorth Parameters are applied to the activity, mobility, and complexity of data, while Higuchi Method describes their fractal dimension; the calculation of entropy and energy is reached thanks to the Wavelet Transform (Al-Nafjan et al., 2017).

Although attention to EEG signals is often predominant in the literature, the possibility of obtaining information from other physiological signals is nevertheless largely described: in multimodal approaches, the response is given by merging various features from different sources, and the performance in emotion recognition is usually higher than unimodal approaches (which have instead more computational advantages; Hasnul et al., 2021).

Hasnul et al. (2021) focus their review on ECG, describing the modalities of analysis applied to extract affective inferences. Data preprocessing is mostly performed through a multiple-configuration Butterworth filter, while feature extraction involves the calculation of statistical features and the detection of beats’ shape and timing. The ML classification is then performed through deep learning or *via* a feature selection, dimensionality reduction, and supervised algorithms (the most frequent is SVM, but also k-nearest neighbor and naïve Bayes are reported to obtain good performances). They also list the affective databases, which include ECG data, available for research purposes; AuBT, AMIGOS, and DREAMER are reported as the most popular in studies involving ECG (Hasnul et al., 2021).

An open and under-developed field is the integration in the day-to-day life of affective detection, also targeted at the development of long-term experimental designs: wearable devices able to detect in a non-invasive way the user’s physiological data may answer the problem. Schmidt et al. (2019) take stock of the situation illustrating that ECG is the most frequent signal applied in wearable-based research, followed by SC (respectively in 87 and 76% of the reviewed studies); the evaluation of respiration, EMG, temperature, and movement (through 3-axes acceleration sensors) are instead presented in 32% of the articles. The studies conducted in the field involved both pre and post-questionnaires (e.g., assessing personality traits or mental health) and different modalities of the Ecological Momentary Assessment (asking the users about their emotional state with a specific frequency, randomly or event-triggered). Data analysis, after the feature extraction, is mostly performed with ML classification; also in this review SVM is reported as the most frequent algorithm, used in almost half of the cases (Schmidt et al., 2019). An interesting description of usage and problematics of EEG wearable devices can be found in Anders and Arnrich’s (2022): regarding the hardware, the literature shows a majority of custom-built devices, followed by products from the InteraXon Muse series, and EMOTIV Epoc, all with a sampling rate of at least 256 Hz. Given the necessity of wearable mobile instruments, special attention needs to be given to the power supply, generally limited, which influences data transmission and storage; these limitations in resources cause restrictions in the number of manageable channels and make it preferable to pre-process data directly on the recording device or even feed them through pre-trained algorithms. Also noise, caused movement will decrease the quality of the obtained recordings. Especially if feedback is wanted in real-time, the temporal analysis should be carefully considered: the most reported are one-second windows, followed by time spans between 4.5 and 30 s (Anders and Arnrich, 2022).

### 4.3. Brain-Computer Interface (BCI)

Brain-Computer Interface is defined as a control and/or communication system that does not depend on normal neuromuscular output channels (Birbaumer, 2006; Nicolas-Alonso and Gomez-Gil, 2012). The user’s intention is recorded through brain signals, depending on muscles or peripheral nerves (Schalk et al., 2004). In the processing of data from the brain, the subject, and machine interact in real-time, allowing the user of this technology to receive immediate feedback from the computer, which translates the data into actions based on the subject’s intent (Wolpaw et al., 2002). This permits the person to appropriately modify their intentions, thanks to a “mental strategy” learned through operating conditioning training, and, therefore, the data that the machine will receive next (Birbaumer, 2006; Graimann et al., 2010).

The computer meanwhile learns to associate the person’s brain activity with the correct command thanks to ML algorithms—mainly classification algorithms (Birbaumer, 2006; Nicolas-Alonso and Gomez-Gil, 2012) - building the human-computer interaction that is at the core of the BCI system (Krepki et al., 2007; Graimann et al., 2010). BCIs can be based on different signal acquisition techniques, such as magnetoencephalography (MEG), functional magnetic resonance imaging (fMRI), Functional Near infrared Spectroscopy (fNIRS) and EEG signal detection. Even though fNIRS is a relatively new modality for detecting brain activity, it has several advantages, including greater portability/safety when compared to fMRI, and lower susceptibility to electrical noise when compared to EEG and MEG (Herff et al., 2013; Hong et al., 2015; Naseer and Hong, 2015; Noori et al., 2017). fNIRS measures the concentration changes of oxygenated hemoglobin (HbO) and deoxygenated hemoglobin (HbR) in blood flow caused by neuron firings in the local capillary network using near-infrared light. The primary motor cortex (associated with motor imagery tasks) and the prefrontal cortex (associated with cognitive tasks such as arithmetic ones, emotion induction etc.) are the most commonly used brain areas in fNIRS-BCI systems (Herff et al., 2013; Hong et al., 2015; Naseer and Hong, 2015; Noori et al., 2017). Despite the introduction of fNIRS-BCI systems and hybrid EEG-fNIRS BCIs (Khan et al., 2020; Liu et al., 2021), EEG-based BCIs alone is more common, since it has lower costs and has greater ease of use compared to the other signals detection techniques (Graimann et al., 2010; Nicolas-Alonso and Gomez-Gil, 2012).

Brain-Computer Interface in which the user intention is substantial for reaching the aim of the application can be defined as “active BCI”; instead, when the system automatically detects specific mental patterns or activations to give feedback or control devices, can be called “passive BCI.” Following this logic, Affective Computing implying EEG inputs fall into the category of “passive BCI” (Al-Nafjan et al., 2017). The definition of “reactive BCI” is also reported, concerning the detection of the automatic brain reactions to a stimulus (e.g., with Event-Related Potentials; Hramov et al., 2021).

To proceed with the recording, the closer the source the electrodes are positioned, the clearer the recording will result in less background noise. At the same time, the placement of very deep electrodes develops medical and safety issues (Wolpaw et al., 2002). In this article, we will focus only on biosensors on the scalp but invasive BCI systems based on Electrocorticography (ECoG), or intracranial electroencephalography (iEEG), where the electrodes are lying on the cortical surface or *via* Intracortical neuron recording (Nicolas-Alonso and Gomez-Gil, 2012; Kandel et al., 2021), are also described in the literature as experimental applications suitable for extreme cases (Birbaumer, 2006; Graimann et al., 2010).

The BCI systems based on EEG are divided depending on the logic applied and the resulting type of waves that are detected to work (Wolpaw et al., 2002; Schalk et al., 2004; Graimann et al., 2010; Nicolas-Alonso and Gomez-Gil, 2012):

- *Event-Related Potential* (ERP; e.g., P300), Visual Evoked Potentials (VEPs), and Slow Cortical Potentials are specific-shaped waves detected after a standard quantity of time (ms) after the presentation of specific stimuli.
- *Sensorimotor rhythms* are divided into the frequencies of 8–12 Hz (mu wave) and 18–26 Hz (beta wave). They are recorded above the sensorimotor cortex and associated with movements and sensations, real and imagined. The control of their amplitude, even in the absence of movements and sensations can be trained and used as output.

Aggarwal and Chugh (2022) review the studies published after 2009 concerning ML and BCI based on EEG, finding that 44% of the studies are based on Motor Imagery, 37% on P300, and 19% on SSVEP (Aggarwal and Chugh, 2022).

**Figure 4:**
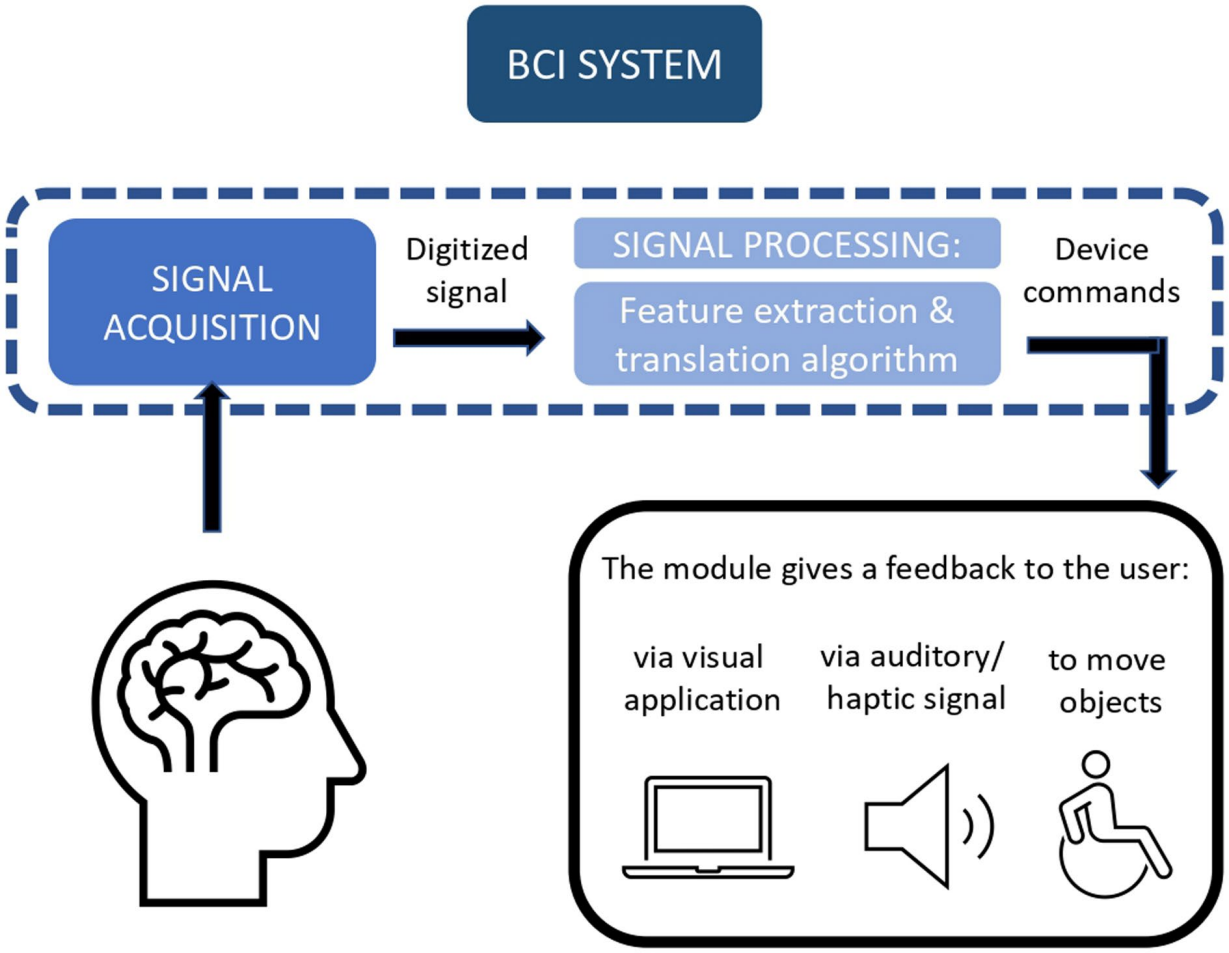
Brain-Computer Interface (BCI) system allows a loop communication between the brain and the computer.

After the signal acquisition is performed, data is digitized and processed. Signal processing is composed of a feature extraction phase and the translation algorithm: in the feature extraction, only specific data are selected, to decrease the computational cost, while the translation algorithm consists of a ML algorithm, which translates, as the name implies, data into an output suitable command devices (Wolpaw et al., 2002; Schalk et al., 2004; Graimann et al., 2010; Nicolas-Alonso and Gomez-Gil, 2012; Figure 4).

For example, Schalk et al. (2004) describe how they have implemented BCI2000, an open-source suite of software for BCI frameworks. Its structure follows the modules necessary for processing a BCI stream of data, starting from an EEG, to become an input for a human-computer interface. The source module is deputed to digitize the brain’s data and memorize data, with eventual event markers. In the signal processing module, feature extraction is performed through linear transformations, which convert the input into microvolts and create a spatial and a temporal filter, while the proper translation algorithm consists of two linear transformations, a classifier, and a normalizer.

After that, the user application module uses the data received from the former module to send feedback to the user: this could be embedded in a visual application, or be presented as an auditory or haptic signal, or move a prosthesis. As last, the operator module allows the caregiver to supervise and modify parameters thanks to a graphical interface. Described use cases include navigation with the computer cursor, use of audio–video, and spelling applications (Schalk et al., 2004).

The use of ML techniques in BCI frameworks, are described in Nicolas-Alonso and Gomez-Gil (2012), in which are listed as algorithms for features extraction and selection techniques PCA, Independent Component Analysis, AutoRegressive Components, Matched Filtering, Wavelet Transform, Common Spatial Pattern, Genetic Algorithm, and Sequential Selection. The most frequent classificators reported are Bayesian analysis, pLDA, SVM, k-NN, and Artificial NNs; furthermore, are described some regression approaches, rather than classification (Nicolas-Alonso and Gomez-Gil, 2012).

As state-of-the-art nowadays, Chamola et al. (2020) reviewed studies on BCI based on EEG, ECoG, and Near-Infrared Spectroscopy (NIRS). The learning paradigms described employed to achieve the BCI goal are all classifiers. As most frequent algorithms reported are pLDA, SVM, Artificial NNs, and Statistical classifiers (Chamola et al., 2020).

Similarly, Cao (2020) reviews ML implementation for EEG-based BCI: classification is described also here as the fundamental learning strategy for BCI, with linear SVM and pLDA algorithms as the best performers. Various kinds of learning are nevertheless finding their place alongside these more traditional ML approaches: Deep Learning (DL) approaches (i.e., NNs, recurrent NNs, and generative adversarial networks) are indeed used in the EEG extraction phase, and then combined with more classic ML algorithms (Cao, 2020).

Aggarwal and Chugh (2022) focalize the use of ML algorithms divided by type of input. In BCI commanded with motor imagery, SVM performs a better classification (two-class or multi-class) than LDA and k-NN (with more computational costs); in P300-BCI, LDA (especially StepwiseLDA and Regularized discriminant analysis) has better results than SVM, alongside with the deep learning model Convolutional neural networks (CNN). Also in SSVEPs applications, SVM is the most frequent algorithm followed by LDA and k-NN, while CNN usage is rising (Aggarwal and Chugh, 2022).

Alzahab et al. (2021), focus on the passage in BCI implementation from more classic ML systems to a combination of DL algorithms, called hybrid DL (hDL): EEG remains the most frequent data recording source, which typically demanded a mandatory complex pre-processing phase. With the introduction of hDL, the pre-processing phase results were performed by just 21.28% of studies, demonstrating how this kind of learning mode could overcome EEG’s Signal-to-Noise Ratio: moreover, the utilization of temporal features for BCI with hDL reach 93.94% accuracy. The most common algorithm reported is Convolutional NNs-Recurrent NNs. In addition, the implementation (in half of the reviewed studies) of DL systems with a low number of layers, diminishing both complexity and computational costs, opens up further future potential applications (Alzahab et al., 2021).

Despite the improvements due to advances in instrumentation and algorithms, some downsides still need to be considered. For example, Hramov et al. (2021) consider (1) slow data transfer, which still makes feel the feedback unnatural, (2) an excess of false positives, (3) an essential need for assistance for the user (especially for physically impaired people), (4) the impossibility of starting the BCI system with a BCI signal (also when shutdown through BCI is available), (5) a high cognitive load need for the BCI control, which pairs with the need of a non-distracting environment, and (6) the lack of standardization among the device models (Hramov et al., 2021).

One of the main deputy areas of application for BCI is the communication enabling of subjects with severe muscular or neurological damages, like Amyotrophic Lateral Sclerosis (ALS; Birbaumer, 2006; Nicolas-Alonso and Gomez-Gil, 2012). An example is the Hex-O-Spell interface, which uses a paradigm similar to the Berlin BCI (BBCI) project. Hex-O-Spell, presented in Blankertz et al. (2007), permits, classifying the sensorimotor rhythms of two different imagined movements (e.g., right hand and right foot movements) to rotate an arrow pointing to six hexagons. Each hexagon contains a letter group and leads to another group of hexagons, one for each letter; the layout of the next hexagon is re-ordered, following a probability distribution, which permitted some prediction of the next choice (Blankertz et al., 2007). The BBCI framework is described in Krepki et al. (2007): the EEG recording is performed on healthy subjects, who completed multiple trials of right and left-hand movements, both imagined, and executed. EEG data were then preprocessed, windowed, and filtered through a Fast Fourier Transformation and pass-band filters, regularized by the Regularized Fisher Discriminant, and divided into time intervals. Each interval was labeled corresponding to the class of the movement left/right, and the rest state. A final classification with 10-fold cross-validation was implemented *via* SVM and linear programming machines. The final command is sent to the human-interface device, corresponding to the class resulting from the classification (left/right/rest; Krepki et al., 2007).

The second aim which leads the study and realization of BCI systems is linked to physical rehabilitation, with solutions related to motor rehabilitation (Birbaumer, 2006; Nicolas-Alonso and Gomez-Gil, 2012; Juliano et al., 2020) or studies on implementing motor aids, such as walking aid (King et al., 2013) or prosthetic (Muller-Putz and Pfurtscheller, 2008) for people with spinal cord injury.

Saha et al. (2021) review how a BCI, giving feedback to the patient (acting as a neurofeedback device) is hypothesized to improve the neuroplasticity (the cortical–subcortical neural networks reorganization) and cognitive rehabilitation, assisting the user to self-regulate specific brain rhythms (Saha et al., 2021).

Leeb et al. (2007) describe the application of a BCI system to let a spinal cord-injured person navigate in a Virtual Reality (VR) Cave space. The driving inputs were extracted from beta oscillation caused by imagined movement of feet, following the Graz-BCI paradigm. The aim of the simulation was to travel along a street, stopping by certain avatars; the subject reached a performance of 100% after four runs (Leeb et al., 2007). The Graz-BCI bases its functionality on the classification of motor imagery (e.g., right-hand versus left-hand movements). This purpose is achieved by recording the EEG activity of a subject repeatedly imagining the intended movements. After that, a feature extraction phase is performed by calculating the EEG band power and applying adaptive autoregressive parameters, and common spatial filters. The translation algorithm is modeled with a neural network and a linear discriminant analysis (Pfurtscheller et al., 2000).

Brain-Computer Interface for simulated motion is also present in the brain-controlled wheelchair by Rebsamen et al. (2010), in which a P300 detection system is implemented to select a destination among pre-definite locations in a VR house (Rebsamen et al., 2010).

Muller-Putz and Pfurtscheller (2008) tested instead the use of a two-axes-moving hand prosthesis, designed for spinal cord-injured people, on four healthy subjects. The selected modality implemented steady-state VEPs (SSVEPs) to discriminate four classes of commands (left, right, open, and close). The “frequency-coded selection by SSVEPs” (Gao et al., 2003) was implemented through an array of LED targets flickering at different frequencies. Staring at one specific LED produces SSVEPs in the brain, with a frequency equal to the flickering frequency of the target. The classification learning resulted in an accuracy of between 44 and 88% (Muller-Putz and Pfurtscheller, 2008).

The last aims of a BCI device are interaction with and control of the environment, and entertainment (Nicolas-Alonso and Gomez-Gil, 2012). In this context, Gao et al. (2003) produced a prototype of a remote controller, which could handle 48 target functions for interfacing with household appliances. The system is based on SSVEP, chosen for its transfer rate level and ease of use. In detail, the “frequency-coded selection by SSVEPs” was implemented, with an array of LEDs on a 6 × 8 visual panel, connected to an infrared remote controller. 87.5% average accuracy was reached, with an average selection time of 3.8 s (Gao et al., 2003). Other types of support to daily life activities are represented by the possibility of using a cursor to interact with specifically implemented graphic interfaces: Vaughan et al. (2006) used both sensorimotor rhythms and P300 to permit the movement of a cursor in two dimensions (Vaughan et al., 2006), and similar outcomes were obtained with a combination of P300 and Mu/Beta rhythm in motor imagery (Li et al., 2010); additionally, even one of the earliest applications of the BBCI is the control of a Pacman video game, intended just for entertainment (Krepki et al., 2007).

Interaction and control of robots could also aid interaction between the impaired person and the environment, and research is focusing on such applications, despite some safety concerns (Cao, 2020). Grasping, telepresence, communication, and navigation in interesting environments (e.g., museums) are also possible practical applications of BCI systems (Chamola et al., 2020).

Brain-Computer Interface caused a lot of enthusiasm in the ‘00s, but despite the technological improvements of recent years, this trend seems to have reversed. This should cause concerns about their actual functionality and practicality for people usually with severe disabilities: the focus on the final user and on the cost–benefit ratio of new technologies is often lacking in the design phases, which may bring researchers even farther away from the patient bedside.

## 5. Challenges in ML and future scenarios

Machine Learning offers several benefits to clinical practice, but it is not exempt from possible drawbacks, some of which are made evident from the ethics perspective. Firstly, ML algorithms used to analyze biosignals represent a mediator between clinicians’ decision-making and patients, raising some concerns related to the transparency of the information provided to both the patient and the clinician. Given the structural complexity of some algorithms (e.g., DNNs), adoption of these tools (and of their predictions) does not allow for a complete understanding of the processes that take place within their hidden layers, not allowing for a full disclosure of information relating to patient care, and thus undermining the clinician’s full decision-making autonomy (Vellido, 2020). Secondly, the exponential increase in the use of digitized sensitive data is not accompanied by a parallel development of laws and regulations for sensitive Big Data use, and this creates some issues related to privacy, security, and informed consent (Floridi et al., 2019; Tsamados et al., 2022). There is also a lack of proper infrastructure for data acquisition and storage (Hossain et al., 2019; Jacobson et al., 2020; Kaissis et al., 2020). Lastly, patients and healthcare providers frequently do not trust the use of artificial intelligence models in a medical/clinical setting due to a lack of adequate training or precise information about ML algorithms’ functioning (Asan et al., 2020; Grote and Berens, 2020). Furthermore, without specific guidelines, healthcare specialists risk overreliance on AI predictions, thus possibly leading to a lower assumption of responsibility (Riva et al., 2022).

To overcome these problems, seven essential factors for building ethical AI have been ideated (Floridi et al., 2020): (i) falsifiability and incremental deployment; (ii) safeguards against the manipulation of predictors; (iii) receiver-contextualized intervention; (iv) receiver-contextualized explanation and transparent purposes; (v) privacy protection and data subject consent; (vi) situational fairness; and (vii) human-friendly semanticization. Similarly, Vayena et al. (2018) emphasize that in order to earn patient trust and provider adoption, ML systems should fully align with the following requirements: (i) data sourcing should comply with privacy and security requirements; (ii) ML system diffusion should meet transparency requirements, and (iii) algorithm training and validation should respect fairness requirements (Vayena et al., 2018).

According to Vellido (2020), to contribute to the diffusion of ML tools in health settings, it is critical to focus on strategies for improving data and model visualization, as well as involve clinical professionals in the design of data analysis and interpretation strategies (Vellido, 2020).

Indeed, clinical professionals can bring their sectoral expertise in all the phases of the ML process, optimizing it: they can collaborate on the creation of a dataset that is as free of bias as possible, and truly representative of the characteristics of the whole clinical population of interest. Poor data selection, due to biases, bad examples, and labels inaccuracy, might lead to the “garbage in, garbage out” phenomenon and poorly working models. When it comes to algorithms applied to the population, for example, cases have been reported in which the sample selection underrepresented or disadvantaged women and non-white people (Dieterich et al., 2016; Intahchomphoo and Gundersen, 2020), posing a wide range of ethical fairness issues (Ntoutsi et al., 2020). Furthermore, in mental health, large datasets required for good AI predictions are rare, and a high drop-out rate hinders the collection of longitudinal data: this could result in a non-representative set of data (Bickman, 2020). In clinical and psychiatric notes, for example, statistically significant differences in prediction errors by race, gender, and insurance type have been discovered (Chen et al., 2019). If supervised models can incorporate human biases from human-chosen data, unsupervised models and black-box systems may produce other types of errors, of unknown nature. In the former case, bias-aware data collection is possible, and models can adhere to ontologies that describe specific data. In the latter case, detecting biases or data noise in non-interpretable models is more complex: AI decisions can only be evaluated retroactively, *via* explainable AI systems (Ntoutsi et al., 2020).

In scientific research, especially in fields such as medicine and mental health, explanations of AI behaviors are needed also for the advancement of science and the transparency of discovery, besides their trustworthiness (Guidotti et al., 2018). For example, in Sturm et al. (2016) a DNN classifier’s decision linked to motor imaginary BCI is explained by transforming the results into a relevance heat map. Through the explanation technique of Layer-wise relevance propagation, “an explanatory layer” is offered to show the EEG activity on the scalp (Sturm et al., 2016). Furthermore, Haufe et al. (2014) demonstrated that noise in the data does not allow a direct neurophysiological interpretation of the information combined from different EEG channels and decoded as BCI inputs. However, explanation techniques such as transforming backward models (which extract latent factors from observed data) into corresponding forward models (which express data as a function of hidden variables) may improve interpretability (Haufe et al., 2014).

Failing to account for racial, gender, and sex differences will result in poor quality results, misjudgments, and discriminatory outcomes (Cirillo et al., 2020). For example, several studies highlight gender differences in emotional psychophysiological responses (Dimberg and Lundquist, 1990; Bianchin and Angrilli, 2012) and stress reactivity (Schmaus et al., 2008). Not accounting for these differences during dataset preparation (and thus not inserting as many female pattern examples as male ones) may lead to erroneous emotional and stress detection. Another step in which clinical professionals have an important responsibility is the interpretation of the ML forecasts. In this phase, it is important that the clinician has clearly understood (where possible) the analyses performed by the algorithm, to provide the patient with medical assistance based on clear, precise, and detailed information. Moreover, in this step it is pivotal to employ all empathic communication skills, bearing in mind that the patient may not understand how these technologies work and may not trust the final prediction.

More technical drawbacks are instead related to the lack of generalizability of ML models, often due to missing data, small sample sizes, and heterogeneity of the dataset (Zhou et al., 2017; Thomas et al., 2020). As explained by Thomas et al. (2020), missing data could be the result of several factors such as errors in human data entry, malfunctioning of the measuring instruments, issues affecting the collection or the processing of the data, and drop-out of participants at follow-up. The small sample size of the datasets is instead due to the fact that in the mental health discipline there are only a few consortia that collect data on patients with neurological and psychiatric problems, and, in most cases, small centers that intend to use ML algorithms have few subjects available to create their own datasets (Thomas et al., 2020). Small sample size in turn might lead to other problems (e.g., overfitting, if the model performs well in the training phase but not with unseen data). To overcome small sample datasets, it is possible to aggregate data from diverse sources/sites, but this brings another critical issue, namely, heterogeneity. A heterogeneous dataset is created if different information (sometimes unknown) is included in the dataset. This information might be related to different inclusion and exclusion criteria, different tools used to collect data (e.g., scanners, questionnaires), different procedures or parameters used in the data collection, and different preprocessing pipelines (Thomas et al., 2020). The importance of the heterogeneous data for the learning task may vary. As a result, concatenating all of the features and treating them all equally important is unlikely to result in optimal learning outcomes (Zhou et al., 2017).

In mental health, the use of ML models is connected to another important technical issue. In many cases, when data from patients are collected to train a ML model, some other “unintentional” information is included (e.g., physiological noise, unknown noise), and this might prove confounding. As stated by Bzdok and Meyer-Lindenberg (2018), **“the prediction performance becomes inflated if the training data used for model fitting and the testing data are somehow statistically dependent, even if they are contaminated in subtle ways.”** The role of other comorbidities that typically patients have, that can affect in a similar way the precision and accuracy of ML models remains to be explored (Bzdok and Meyer-Lindenberg, 2018).

In the future, we might expect interesting integration scenarios between computational intelligence and other tools capable of collecting biosignals (and not) in a multimodal manner. The emerging VR technology in healthcare may indeed allow the collection of various information, thanks to sensors built into the viewer and sensors which can be placed on an individual’s body during a VR assessment (Riva et al., 2019). Additionally, recent trends in the rising use of telemedicine systems (particularly after the pandemic) indicate that an increasing number of people will receive remote medical assistance in the future. This implies the need for researching the best ways to provide medical and psychological services remotely, with a focus on how to maximize the benefits of the integration of various devices (apps, smart watches, etc.) to address the patient’s individual needs.

## 6. Conclusions

This article illustrated how mental health disciplines can benefit from the applications of certain branches of Computational Intelligence, such as ML. The most commonly used biosignals in psychology and mental health were firstly described, along with their most interesting components, and relative psychological correlates.

The fundamental phases of ML were defined: pre-processing (comprehending in turn data cleaning, reduction, and transformation), training, validation, and testing. It is critical that each of these steps is completed accurately in order to avoid the model being towed on a dataset that is too small, has implicit bias, or has missing data/errors, that would prevent the model from generalizing the forecasts. Furthermore, the ML training phase was described in detail in terms of which kind of learning algorithms can be used according to specific research questions/tasks and available data information. Indeed, we can distinguish supervised, unsupervised, semi-supervised, and reinforcement learning algorithms, with varying degrees of freedom on the amount of “supervision” and teaching they receive when learning the patterns from the initial dataset (as well as other differences in shape and structure). Validation and test phases were also illustrated, to underline how they are reliable for generalization and to avoid overfitting.

Despite the discussed drawbacks, ML application to mental health fields and biosignals interpretation is desirable since it has the potential to produce more reproducible and generalizable results in neuropsychological research (Breiman, 2001; Bzdok and Meyer-Lindenberg, 2018). This is possible because ML allows complex and multi-modal data elaboration which leads to probabilistic results, expanding current understanding of the human mind, physiology, and behavior, as well as being beneficial for the personalization of the mental health field. For this latter point, clinical Big Data (analyzed with ML) will be increasingly bent to serve individuals’ needs through Precision Medicine, an approach to healthcare that uses technology to find appropriate individual solutions to clinical conditions, resulting in more humane treatments for complex or rare conditions (Cipresso and Immekus, 2017; Orrù et al., 2019; Rutledge et al., 2019; Tuena et al., 2020).

Machine Learning also allows cutting-edge clinical applications like Affective Computing and BCIs. Affective Computing applies ML models to interpret biosignals and extrapolate information about emotional states: breathing, ECG, BVP, SC, and facial muscles EMG can be processed through supervised learning classification models for categorizing emotional arousal and valence. BCI allows the creation of a human-computer real-time loop that returns the user feedback (*via* computer) that translates into actions based on the user’s intentions. Since its beginnings, BCI has relied heavily on supervised ML models for EEG signal analysis; however, its performance is improving thanks to the increasing use of DL algorithms. Applications in mental health range from allowing patients with movement disorders to move a wheelchair, or people with speech impediments to communicate *via* computer. We have seen that integration between BCI, and affective computing is still today a little explored area of medical sciences, even if some studies have been conducted in the fields of Biofeedback or Neurofeedback training.

## Author contributions

ES, GR, CT, and PC contributed to the conception and design of the study. ES wrote the first draft of the manuscript. ES and SB wrote sections of the manuscript. SB conceived and produced the images. All authors contributed to the article and approved the submitted version.

## Funding

Research funded by the Italian Ministry of Health (POSTECH: 39C801_2018).

## Conflict of interest

The authors declare that the research was conducted in the absence of any commercial or financial relationships that could be construed as a potential conflict of interest.

## Publisher’s note

All claims expressed in this article are solely those of the authors and do not necessarily represent those of their affiliated organizations, or those of the publisher, the editors and the reviewers. Any product that may be evaluated in this article, or claim that may be made by its manufacturer, is not guaranteed or endorsed by the publisher.
